# Immunomodulatory Effects Associated with Cladribine Treatment

**DOI:** 10.3390/cells10123488

**Published:** 2021-12-10

**Authors:** Nicolás Fissolo, Laura Calvo-Barreiro, Herena Eixarch, Ursula Boschert, Carmen Espejo, Xavier Montalban, Manuel Comabella

**Affiliations:** 1 Centre d’Esclerosi Múltiple de Catalunya (Cemcat), Institut de Recerca Vall d’Hebron (VHIR), Hospital Universitari Vall d’Hebron, Universitat Autònoma de Barcelona, 08035 Barcelona, Spain; lauracalvobarreiro@gmail.com (L.C.-B.); herena.eixarch@vhir.org (H.E.); carmen.espejo@vhir.org (C.E.); xavier.montalban@cem-cat.org (X.M.); 2 Ares Trading, SA, 1262 Eysins, Switzerland; ursula.boschert@merckgroup.com; 3 Merck KGaA, 64297 Darmstadt, Germany

**Keywords:** multiple sclerosis, cladribine, immune-regulation, flow-cytometry

## Abstract

Cladribine is a synthetic deoxyadenosine analogue with demonstrated efficacy in patients with relapsing-remitting multiple sclerosis (MS). The main mechanism of action described for cladribine is the induction of a cytotoxic effect on lymphocytes, leading to a long-term depletion of peripheral T and B cells. Besides lymphocyte toxicity, the mode of action may include immunomodulatory mechanisms affecting other cells of the immune system. In order to induce its beneficial effects, cladribine is phosphorylated inside the cell by deoxycytidine kinase (DCK) to its active form. However, the mechanism of action of cladribine may also include immunomodulatory pathways independent of DCK activation. This in vitro study was designed to explore the impact of cladribine on peripheral blood mononuclear cells (PBMC) subsets, and to assess whether the immunomodulatory mechanisms induced by cladribine depend on the activation of the molecule. To this end, we obtained PBMCs from healthy donors and MS patients and performed proliferation, apoptosis and activation assays with clinically relevant concentrations of cladribine in DCK-dependent and -independent conditions. We also evaluated the effect of cladribine on myeloid lineage-derived cells, monocytes and dendritic cells (DCs). Cladribine decreased proliferation and increased apoptosis of lymphocyte subsets after prodrug activation via DCK. In contrast, cladribine induced a decrease in immune cell activation through both DCK-dependent and -independent pathways (not requiring prodrug activation). Regarding monocytes and DCs, cladribine induced cytotoxicity and impaired the activation of classical monocytes, but had no effect on DC maturation. Taken together, these data indicate that cladribine, in addition to its cytotoxic function, can mediate immunomodulation in different immune cell populations, by regulating their proliferation, maturation and activation.

## 1. Introduction

Cladribine (2-chlorodeoxyadenosine) is a synthetic deoxyadenosine analogue [1] initially licensed as a chemotherapeutic agent used to treat hairy cell leukemia [2], with demonstrated efficacy in patients with relapsing-remitting (RR) multiple sclerosis (MS) [3,4,5]. Cladribine appears to preferentially affect lymphocytes in a relatively selective manner, leading to a sustained reduction of peripheral T and B lymphocytes and finally, long-lasting lymphopenia [6].

Cladribine is a prodrug that is actively transported into cells via the purine nucleoside transporters. Once inside, cladribine is phosphorylated by deoxycytidine kinase (DCK) to the active triphosphorylated deoxyadenosine [7,8], an action opposed by the phosphorylase activity of cytosolic 5′-nucleotidases (5-NT). In those cells with a high DCK to 5-NT ratio, such as lymphocytes and monocytes, cladribine is phosphorylated to its active form. The accumulation of the phosphorylated molecule to toxic concentrations disrupts cellular metabolism and damages DNA, resulting in cell death [1].

Besides lymphocyte depletion, cladribine may spread its influence to other immune cells. Indeed, it has been reported that cladribine also reduces the number of innate immune cells including monocytes and natural killer (NK) cells, although to a lesser extent than lymphocytes [9,10]. Moreover, cladribine has been associated with a rise in the plasmacytoid to myeloid dendritic cell (DC) ratio [11].

The abovementioned immune regulation has been described as dependent on the activation of cladribine by DCK. However, cladribine may induce immunomodulatory pathways independent of DCK activation, acting as an agonist of adenosine receptors (mainly A1 receptors, but also A2A receptors) [12]. A1 receptors are the most abundant and are expressed in all tissues, having their highest expression in the central nervous system (CNS) [13,14]. A2 receptors are also expressed in the CNS, though their highest expression is found in the striatum, spleen, thymus and immune cells [15]. The overall role of adenosine receptors in the CNS is complex, playing important functions both in physiological and pathophysiological conditions [16]. On the other hand, adenosine receptors have a key role in the modulation of inflammatory processes, inducing anti-inflammatory responses and inhibiting tissue injury caused by inflammation [17]. Altogether, these data point to the potential therapeutic role of the agonism of adenosine receptors by cladribine in the pathogenesis of MS.

The aim of this study was to further investigate the effects of cladribine on peripheral blood mononuclear cell (PBMC) subsets, and to assess whether immunomodulatory mechanisms induced by cladribine depend on the activation of the molecule. To carry out this process, we first determined the functional concentration of cladribine that induced immunomodulation in PBMCs. Second, we evaluated the impacts of cladribine on proliferation, survival and activation of T, B and NK cells in DCK-dependent and -independent conditions. Finally, we examined the effect of cladribine on monocyte activation and DC maturation.

## 2. Materials and Methods

### 2.1. Subjects

Blood donations were obtained from healthy donors and MS patients at the Multiple Sclerosis Centre of Catalonia (Cemcat), Vall d’Hebron Hospital, Barcelona, Spain. Patients with relapsing-remitting MS [18], fulfilling the criteria for treatment with immunomodulatory therapies, and sex- and age-matched healthy donors were included in the study. The Vall d’Hebron Ethics Committee approved this procedure and written informed consent was obtained from every blood donor.

### 2.2. Cell Isolation

PBMCs were isolated using standard Ficoll gradient centrifugation. Monocytes were positively selected from PBMCs using human anti-CD14 microbeads (Miltenyi Biotec, Bergisch Gladbach, Germany) according to the manufacturer’s instructions. To generate standard monocyte-derived DCs (moDCs), isolated monocytes were cultured for 6 days in the presence of 1000 U/mL of granulocyte–macrophage colony-stimulating factor (GM-CSF) and 400 U/mL of IL-4, at 37 °C, 5% CO2 and >95% humidity. Cytokines were refreshed by adding the same volume of starting medium on day 3. After 6 days of culture, moDCs were obtained. For differentiation into mature DCs (mDCs), immature DCs (ImDCs) were also stimulated with 100 ng/mL of lipopolysaccharide (LPS; Sigma-Aldrich, St. Louis, MO, USA) on day 6 for 24 h. The purity of the monocyte fraction and the phenotype of ImDCs and mDCs were checked by flow cytometry, based on the surface expression of CD14 (monocyte marker), CD209 (DC-sign), and CD83 (maturation marker). The list of antibodies and clones used to stain samples is shown in the Supplementary Table S1.

### 2.3. Media and Reagents

Human PBMCs were maintained in RPMI 1640 growth medium (Life Technologies, Carlsbad, CA, USA), supplemented with 1% glutamine, 1% penicillin, 1% streptomycin and 5% fetal calf serum (FCS; Gibco, Carlsbad, CA, USA), hereafter referred to as complete medium. Differentiation of monocytes to ImDCs and further maturation to mDCs was performed with an Mo-DC differentiation medium (Miltenyi Biotec). Cladribine (Merck, Darmstadt, Germany) and deoxycytidine (Sigma-Aldrich) were dissolved in DMSO and water, respectively, and further diluted to the appropriate concentration in complete medium.

### 2.4. In Vitro Stimulation of PBMCs and Functional Assays

Isolated PBMCs were cultured at a concentration of 1 × 10^6^ cells/mL in complete medium at 37 °C, 5% CO2 and >95% humidity on 24-well plates. Depending on the functional assay, cells were stimulated either with phorbol 12-myristate 13-acetate (PMA) plus ionomycin or phytohemagglutinin (PHA) (all from Sigma-Aldrich) and treated with cladribine in the presence or absence of deoxycytidine. When used in combination with cladribine, deoxycytidine was pre-incubated with the cells for 30 min prior to the addition of cladribine.

#### 2.4.1. Proliferation

For the dose–response experiments, the proliferation of whole PBMCs was assessed by the thymidine incorporation assay. To this end, PBMCs were stimulated with PHA (5 μg/mL) and treated with increasing concentrations of cladribine (0.01 μM, 0.1 μM, 1 μM, and 10 μM) or left untreated (control condition) for 24 h. Then, complete medium containing 1 μCi of [^3^H]-thymidine (PerkinElmer, Waltham, MA, USA) was added to each well, and the cells were maintained under the same conditions for an additional 18 h. Then, incorporated radioactivity was measured in a beta-scintillation counter (Wallac, Turku, Finland). Stimulation indices were calculated as the ratios of the mean counts per minute of PBMCs in the different experimental conditions, with respect to untreated samples without stimulation. Proliferation of individual PBMC populations was assessed by measuring carboxyfluorescein succinimidyl ester (CFSE) dilution using flow cytometry. Prior to stimulation and culture, cells were labelled with the intracellular fluorescent dye CFSE (Thermo Fisher Scientific, Waltham, MA, USA) at a final concentration of 1 μM of CFSE, at 20 °C for 5 min. According to the literature, cells need to proliferate for at least 4 days in order to quantify the dividing cells (decrease in CFSE staining); in those conditions, the concentration of PHA was reduced (0.25 μg/mL) and 0.1 μM or 1 μM cladribine, with or without deoxycytidine (250 μM), were added to the culture for only the last 48 h, in order to avoid a high cell-death rate. The proliferation of individual cell subtypes was evaluated after labelling with a panel of monoclonal antibodies (mAbs), as described in the Section 2.6 of the methods.

#### 2.4.2. Apoptosis

For dose–response experiments, PBMCs were stimulated with 5 μg/mL of PHA and treated with cladribine at 0.01 μM, 0.1 μM, 1 μM, 10 μM, or left untreated for 16 h. The experimental conditions for the apoptosis functional assay included PHA-stimulation of PBMCs and treatment with 0.1 μM or 1 μM of cladribine in the presence or absence of 250 μM of deoxycytidine. The detection of apoptotic cells was carried out by flow cytometry, by means of Annexin V/7AAD (BD Biosciences, San Jose, CA, USA) staining. Cell subsets were stained using fluorochrome-conjugated mAbs, as described in the Section 2.6 of the method.

#### 2.4.3. Activation

PBMCs were stimulated with 10 ng/mL PMA plus 500 ng/mL of ionomycin, and treated with 0.1 or 1 μM of cladribine in the presence or absence of 250 μM of deoxycytidine for 4 h. At the end of the cell culture, the activation status (expression of CD69) was determined by flow cytometry. PBMCs were labeled as described in the Section 2.6 of the method.

### 2.5. In Vitro Stimulation of Monocytes and Activation Assay

Monocytes were seeded at a concentration of 1 × 10^6^ cells/mL on 24-well plates. Then, cells were stimulated with 100 ng/mL of LPS, and treated with 0.1 μM or 1 μM cladribine during 24 h. Monocyte activation was assessed by flow cytometry, by means of the expression of CD25, CD80 and HLA-DR (Supplementary Table S1). Cell labelling is described in Section 2.6.

### 2.6. Flow Cytometry

After in vitro stimulation and treatment, PBMCs, monocytes and DCs used on different assays were harvested and stained for flow cytometry analysis. PBMCs were stained with fluorescently labelled antibodies against: CD45 (leukocytes), CD3, CD4/CD8 (T cells), CD19 (B cells), and CD56 (NK cells). Gating strategies for assessing proliferation, apoptosis, and activation of PBMCs are described in the Supplementary Figures S1–S3, respectively. Monocyte subsets were classified according to the expression of CD14 and CD16 (classical CD14^++^ CD16^−^, and non-classical CD14^+^ CD16^++^), and fluorescently labelled antibodies against CD25, CD80, and HLA-DR were used to assess monocyte activation. The gating strategy for the monocyte activation assay is depicted in the Supplementary Figure S4. DC maturation was measured by means of the expression of the lineage markers CD14 and CD209, and the maturation marker CD83. Gating strategy for DC maturation is shown in the Supplementary Figure S5. Except for the apoptosis assay, discrimination of dead cells was achieved by Fixable Viability Stain. Samples were acquired with a CytoFLEX flow cytometer (Beckman Coulter, Brea, CA, USA) and data were analyzed with CytExpert 2.3 software (Beckman Coulter). The list of antibodies and clones used to stain samples is shown on Supplementary Table S1.

### 2.7. Statistical Analysis

Statistical analysis was conducted using SPSS (Version 20) software (SPSS Inc., Chicago, IL, USA) for MS Windows and GraphPad Prism 6.0 software (GraphPad Prism Inc., La Jolla, CA, USA). Data from PBMCs are presented using box and whisker plots; boxes show the median and 25th and 75th percentiles, and whiskers represent the minimum and maximum values. One-way ANOVA with repeated measures following a Dunnett multiple comparisons test was used, taking the control condition (no cladribine) as a reference. Data from the monocytes and DCs are presented in bar graphs with mean values ± standard error of mean (SEM). Following the assessment of normal distribution with the Kolmogorov–Smirnov test, for these experiments a Mann–Whitney test or Student’s *t*-test were applied to compare specific conditions to each other. Significance was defined based on *p*-values (* *p* < 0.05, ** *p* < 0.01, *** *p* < 0.001, **** *p* < 0.0001).

## 3. Results

### 3.1. Assessment of the Functional In Vitro Dose of Cladribine

**Figure 1:**
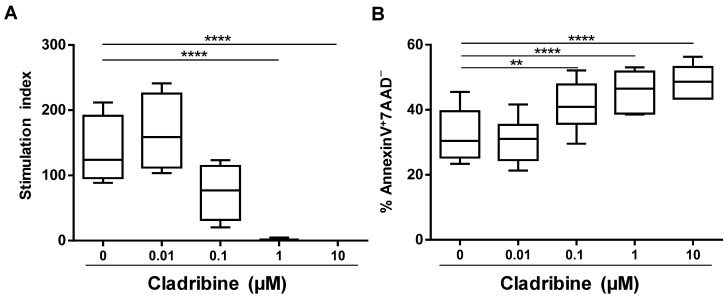
Dose–response effect of cladribine on cell proliferation and apoptosis of PBMCs. (**A**) Proliferation was determined by the incorporation of [^3^H]-thymidine. PBMCs were stimulated with PHA for 42 h in the presence of increasing concentrations of cladribine (0.01 μM, 0.1 μM, 1 μM and 10 μM). Stimulation indices were calculated as the ratios of the mean counts per minute of PBMCs, in the different conditions of culture, with respect to unstimulated and untreated PBMCs. (**B**) Cellular viability was assessed by flow cytometry, by means of Annexin V/7AAD staining on PHA-stimulated PBMCs treated with cladribine at 0.01 μM, 0.1 μM, 1 μM, 10 μM, or left untreated for 16 h. Apoptotic cells were defined as Annexin V^+^7AAD^−^ cells. One-way ANOVA with repeated measures followed by a multiple comparison Dunnett’s test was performed, comparing every mean value with a control of the untreated group mean (0 group). ** *p*-value ˂ 0.01, **** *p*-value ˂ 0.0001. (*N* = 6).

To establish a working-dose of cladribine in vitro, we assessed the effect of cladribine on proliferation (incorporation of [^3^H]-thymidine) and apoptosis (percentage of Annexin V^+^7AAD^−^) of PBMCs as a whole. Unstimulated or PHA-stimulated PBMCs were incubated with increasing concentrations of cladribine ranging from 0.1 to 10 μM. As depicted in Figure 1A, the incubation of cells with 0.1 µM cladribine and higher reduced the stimulation index of the PHA-stimulated PBMCs, compared with the untreated condition, and the differences became statistically significant for the 1 and 10 µM concentrations. Likewise, these three concentrations led to significant increases in the percentage of CD45^+^-leukocytes undergoing apoptosis (Annexin V^+^7AAD^−^), when compared with untreated cells (Figure 1B). Based on these results, the following experiments were performed using the 0.1 and 1 µM concentrations of cladribine. Experimental conditions were selected, taking into account that 0.1 μM was the mean concentration found in the plasma of patients treated with cladribine [19], and 1 μM was the minimum concentration that showed an effect on the proliferation of the PHA-stimulated PBMCs.

### 3.2. Inhibition of Cell Proliferation Caused by Cladribine Depends on Prodrug Activation

**Figure 2:**
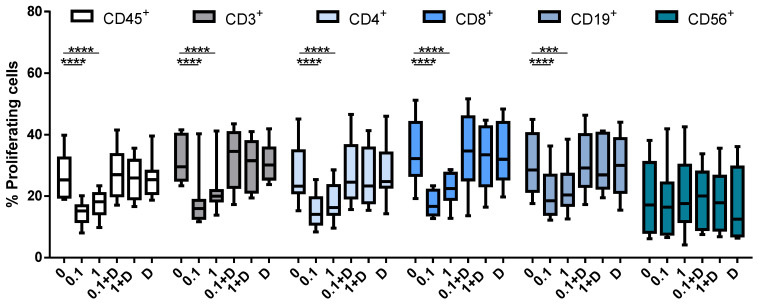
Box plots showing the percentage of proliferating cells in CFSE-stained PBMCs. Cells were stimulated with PHA at 0.25 μg/mL for 96 h. No cladribine, cladribine at 0.1 μM or 1 μM in the presence or absence of 250 μM of deoxycytidine was added for the last 48 h. Percentages of gated CD45^+^, CD3^+^, CD4^+^, CD8^+^, CD19^+^ and CD56^+^ that lost basal CFSE basal expression are shown. The gating strategy for this experiment is shown in Supplementary Figure S1. One-way ANOVA with repeated measures followed by Dunnett’s multiple comparisons test was performed comparing every mean value to a control mean (0 group). *** *p*-value ˂ 0.001, **** *p*-value ˂ 0.0001. (*N* = 10). Cladribine concentrations (μM) are plotted on the x axis. D: deoxycytidine.

The next step was to determine which cell subtypes within PBMCs are affected by cladribine in vitro, and whether this effect depends upon activation by DCK. To determine the effect of cladribine on cell proliferation, we performed a CFSE staining on PBMCs prior to PHA stimulation and treatment with 0.1 or 1 µM of cladribine. In addition, to study whether cladribine acts in a DCK-independent manner, cells were pre-treated with 250 μM of deoxycytidine for 30 min, a molecule that competes with cladribine for the phosphorylation via DCK. Proliferation of PBMCs, stimulated with PHA was impaired by cladribine in leukocytes (CD45^+^), T cells (CD3^+^CD4^+^CD8^−^ and CD3^+^CD4^−^CD8^+^), and B cells (CD19^+^), but not in NK cells (CD3^−^CD56^+^) (Figure 2). On the other hand, cladribine did not have any effect on proliferation when it was combined with deoxycytidine, since the percentage of cell proliferation was comparable with the untreated condition (Figure 2). These results suggest that cladribine is exerting its inhibitory effect on PBMC proliferation in a DCK-dependent manner.

### 3.3. Cladribine Induces Apoptosis in a DCK-Dependent Manner

**Figure 3:**
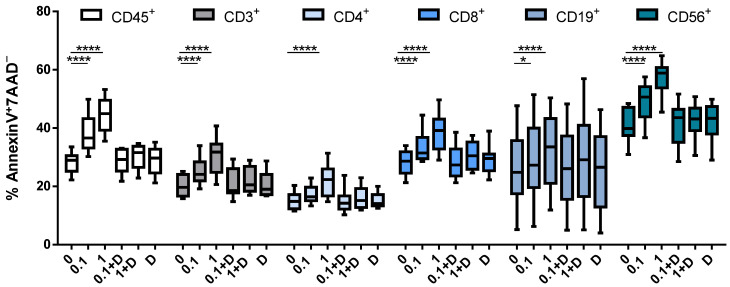
Effect of cladribine on apoptosis of PBMCs. Isolated PBMCs were stimulated with 5 μg/mL PHA and treated with no cladribine, 0.1 or 1 μM of cladribine, in the presence or absence of 250 μM of deoxycytidine, for 16 h. Percentages of gated CD45^+^, CD3^+^, CD4^+^, CD8^+^, CD19^+^ and CD56^+^ that show positivity for Annexin V and negativity for 7AAD are represented. Gating strategy for this experiment is shown in the Supplementary Figure S2. One-way ANOVA with repeated measures followed by Dunnett’s multiple comparisons test was performed, comparing every mean value to a control untreated group mean (0 group). * *p*-value ˂ 0.05, **** *p*-value ˂ 0.0001. (*N* = 10). Cladribine concentrations (μM) are plotted on the x axis. D: deoxycytidine.

We then assessed the effect of cladribine over cell viability. To this end, apoptosis was monitored by the Annexin V/7AAD staining, as previously described. Briefly, PBMCs were stimulated with PHA and treated with the selected doses of cladribine (0.1 or 1 μM), with or without an excess amount of deoxycytidine (250 μM). As shown in Figure 3, treatment with cladribine led to a significant increase in the percentage of cells undergoing apoptosis. A significant dose effect in early apoptosis (Annexin V^+^7AAD^−^) was observed in total leukocytes (CD45^+^), whole T cells (CD3^+^), and CD3^+^CD4^+^/CD8^+^ T cells. Cladribine also induced apoptosis in B cells (CD19^+^) and NK cells (CD3^−^CD56^+^). In contrast, the pre-incubation of cells with deoxycytidine abrogated the effect of cladribine over cell viability, again indicating that cladribine induces apoptosis via DCK.

### 3.4. Cladribine Impairs Cell Activation Both Dependently and Independently of Deoxycytidine Kinase Activity

**Figure 4:**
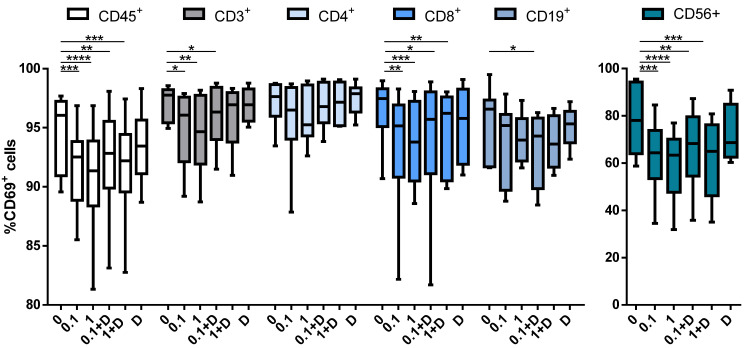
Box plots showing the impact of cladribine on the activation of PBMCs subpopulations. PBMCs were stimulated with PMA plus ionomycin for 4 h with no cladribine (untreated condition), 0.1 μM or 1 μM of cladribine, in the presence or absence of 250 μM deoxycytidine. Activation was represented as the percentage of gated CD45^+^, CD3^+^, CD4^+^, CD8^+^, CD19^+^ and CD56^+^ that showed positivity for CD69. The gating strategy for this experiment is shown in the Supplementary Figure S3. One-way ANOVA with repeated measures followed by Dunnett’s multiple comparisons test was performed, comparing every mean value to a control mean (0 group). * *p*-value ˂ 0.05, ** *p*-value ˂ 0.01, *** *p*-value ˂ 0.001, **** *p*-value ˂ 0.0001. (*N* = 10). Cladribine concentrations (μM) are represented on the x axis. D: deoxycytidine.

In order to explore the effect of cladribine on the activation status of the different immune cell subtypes, PBMCs were stimulated with PMA (10 ng/mL) plus ionomycin (500 ng/mL), and treated with 0.1 or 1μM of cladribine in the presence or absence of 250 μM of deoxycytidine for 4 h; then, activation was measured by flow cytometry, by means of CD69 expression. Cladribine significantly decreased the expression of CD69 in leukocytes (CD45^+^), T cells (CD3^+^), CD8^+^ T cells (CD3^+^CD4^−^CD8^+^) and NK cells (CD3^−^CD56^+^) cells. Conversely, no impact on activation was observed on CD4^+^ T cells (CD3^+^CD4^+^CD8^−^) and B cells (CD19^+^) (Figure 4). Interestingly, in contrast with the impact of cladribine over proliferation and survival, the effect of cladribine on PBMC activation was also present on the same cell populations, following incubation with deoxycytidine (Figure 4). These data indicate that the decrease in immune cell activation by cladribine is not only mediated through phosphorylation of the molecule, but also via alternative pathways not requiring prodrug activation.

### 3.5. Cladribine Shows a Marginal Effect on Monocyte Activation

**Figure 5:**
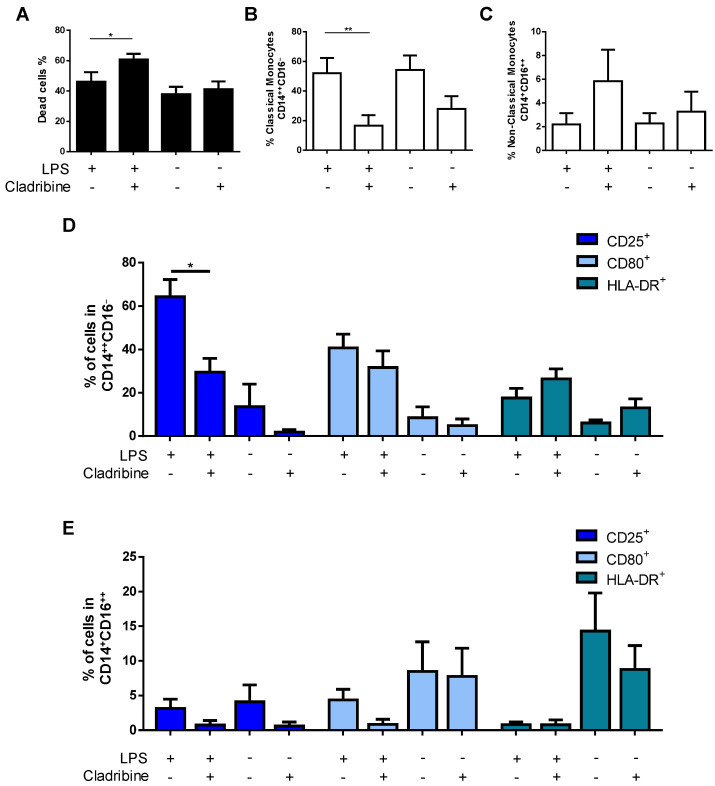
Effect of cladribine on the activation of monocytes. Purified monocytes were treated with 0.1 μM cladribine for 24 h, in the absence or presence of LPS (100 ng/mL), and activation was assessed by flow cytometry, by means of immunostaining against surface activation markers in classical and non-classical monocytes. (**A**) Cell death of total CD14^+^-monocytes was assessed by flow cytometry using a fixable viability dye. After selecting alive cells, monocytes were gated on classical and non-classical monocytes based on the expression of CD14 and CD16. The effect of cladribine in the percentage of (**B**) classical (CD14^++^CD16^−^) and (**C**) non-classical (CD14^+^CD16^++^) monocytes was evaluated. The effect of cladribine over monocyte activation determined in (**D**) classical, and (**E**) non-classical monocytes, is shown as the percentage of the corresponding monocyte phenotype expressing CD25, CD80, and HLA-DR. The gating strategy for this experiment is shown in Supplementary Figure S4. Mann–Whitney tests or Student’s *t*-tests were applied to compare the mean value of LPS + cladribine to a control mean (LPS group). * *p*-value ˂ 0.05, ** *p*-value ˂ 0.01. Data are expressed as the mean. Error bars represent the SEM, (*N* = 5). LPS: lipopolysaccharide.

The activation of myeloid cells such as monocytes has been demonstrated to play a pathogenic role in MS. To investigate the influence of cladribine on LPS-induced monocyte activation, isolated CD14^+^-monocytes were incubated with or without 100 ng/mL of LPS for 24 h, in the presence or absence of the mean concentration of cladribine found in the blood of treated MS patients (0.1 μM). Cell activation was evaluated, measuring the expression of surface activation markers (CD25, CD80, and HLA-DR) in classical (CD14^++^CD16^−^) and non-classical (CD14^+^CD16^++^) monocytes by flow cytometry. Since cladribine has been reported to induce cell death in blood monocytes [10], a fixable viability dye was included to select only alive cells. An induction of cell death by cladribine was observed in LPS-activated cells; however, cell death was similar in non-activated monocytes in the presence or absence of cladribine (Figure 5A). Following the discrimination of dead cells, a significant decrease in the frequency of classical monocytes was observed in the cells treated with cladribine (Figure 5B). Although a slight increase in the frequency of non-classical monocytes was observed in activated monocytes exposed to cladribine, differences did not reach statistical significance (Figure 5C). When the effect of cladribine on the activation status of monocytes was evaluated, a significant reduction in the expression of the activation marker CD25 was observed in classical monocytes, whereas no differences were detected for the CD80 and HLA-DR markers (Figure 5D). Conversely, no differences were observed in any of the activation markers when gated for non-classical monocytes (Figure 5E).

### 3.6. Cladribine Does Not Affect the Differentiation Process of DCs

**Figure 6:**
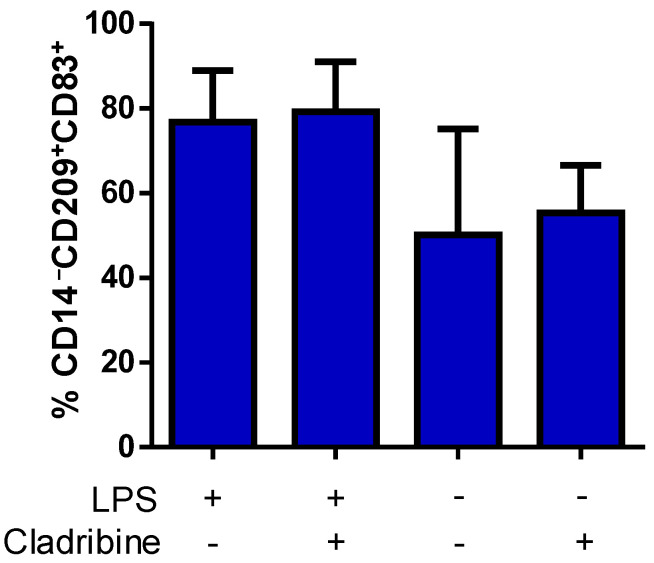
Effect of cladribine on LPS-induced DC maturation. ImDCs were cultured in the presence or absence of 100 ng/mL of LPS, and treated with no cladribine or 0.1 μM cladribine for 24 h. Staining for the maturation (CD83), DCs (CD209) and monocyte (CD14) markers was performed, and alive cells were selected based on the staining with a viability dye. We quantified mDCs in the alive CD14^−^CD83^+^CD209^+^ cells by flow cytometry. The gating strategy for this experiment is shown in the Supplementary Figure S5. Mann–Whitney test or Student’s *t*-test were applied to compare the mean value of LPS + cladribine to a control mean (LPS group). Values represent mean ± SEM, (*N* = 5). LPS: lipopolysaccharide.

Aiming to examine the potential immunomodulatory action of cladribine on DCs, we investigated the effect of cladribine on the maturation of DCs. ImDCs were treated with cladribine (0.1 µM) in the presence or absence of LPS (100 ng/mL) for 24 h. Then, cells were harvested and stained with fluorochrome-conjugated mAbs against the maturation marker CD83, and the lineage markers CD209 (DC-sign) and CD14 (monocytes). Flow cytometry analysis showed that LPS tended to increase the frequency of mDCs (CD83^+^CD209^+^CD14^−^); however, the addition of cladribine did not show any effect on the maturation process (Figure 6).

## 4. Discussion

In this study, we report comprehensive research regarding the immunomodulatory effects of cladribine on PBMCs. We investigated the impact of in vitro cladribine treatment on the cell proliferation, survival and activation of different immune cell subsets from PBMCs, as well as on monocyte activation and moDC differentiation. We also expanded the existing knowledge about the mode of action of cladribine, by studying the effect of the drug in its inactive form. We found that cladribine reduced the proliferative capacity of T cells and B cells, but not NK cells, in vitro. Cladribine also increased the apoptosis of T cells, B cells, and NK cells in a dose-dependent manner. In addition, exposure to cladribine resulted in a significantly decreased activation of total leukocytes (CD45^+^), total T cells (CD3^+^), and CD3^+^CD8^+^ T cells, and NK cells. Interestingly, the inhibition of phosphorylation by DCK completely abrogated the effect of the drug in proliferation and cell survival, but its impact on immune cell activation remained on the same cell populations, suggesting that other mechanisms might also be involved. Moreover, cladribine reduced the frequency of classical monocytes, as well as the expression of the activation marker CD25. Regarding moDCs, cladribine treatment did not affect the LPS-induced maturation process of these cells.

In order to establish an in vitro functional dose for cladribine, we assessed the effects of increasing concentrations of cladribine on the proliferation and apoptosis of PBMCs. In clinical trials, it was determined that after the administration of a 10 mg tablet of cladribine, the maximum plasma concentration found in patients was in the range of 21 to 29 ng/mL (0.07 to 0.1 µM) [19]. Taking this into account, the concentration of 0.1 µM used in our in vitro study was chosen to reflect the concentration that occurs during in vivo situations. In addition, we used lower (0.01 µM) and higher (1 and 10 µM) concentrations to investigate a potential dose-response effect induced by cladribine. We found that cladribine significantly reduced cell proliferation of PBMCs only at high concentrations (1 and 10 µM). Likewise, when PBMCs were exposed to increasing concentrations of cladribine for 16 h, treatment-induced apoptosis occurred at high concentrations (1 and 10 µM), but also at the mean estimated exposure levels of cladribine (0.1 µM), both effects being dose-dependent. Although the strongest effects reaching statistical significance were observed at high concentrations of cladribine, we decided to include the 0.1 µM concentration as the mean estimated exposure levels of cladribine and 1 µM concentration as the minimum concentration showing an effect on PBMCs in the subsequent experiments of our study.

The administration of cladribine in vitro resulted in a decreased proliferation of total leukocytes, T cells, and B cells. These results are consistent with previous studies showing that cladribine reduced the proliferation of PBMCs and T cells [20]. It is worth mentioning that this effect was unrelated to cladribine-induced cell death, since dead cells were removed from the analysis. On the other hand, cladribine had no impact on the proliferative capacity of NK cells, an effect that has not been addressed in previous reports. The decrease in the proliferative response was paralleled by a strong, dose-dependent, increase in the percentage of early apoptotic cells (Annexin V^+^7AAD^−^) for total leukocytes, T cells, B cells, and NK cells. Previous reports have shown the induction of apoptosis in total PBMCs, T cells [20] and B cells [21] by cladribine in vitro. On the other hand, lymphocyte depletion, leading to different grades of lymphopenia for several months, has been described in patients participating in clinical trials with cladribine [22]; however, this lymphotoxic effect has not been quantified as apoptosis.

Regarding cell activation, PMA/ionomycin-induced PBMC activation measured by CD69 expression was inhibited by cladribine in leukocytes, CD8^+^ T cells, and NK cells. Interestingly, when cells were pretreated with an excess amount of deoxycytidine, a competitive substrate for DCK-dependent phosphorylation, no influence on cladribine-induced effect was observed. In this regard, a previous study showed that this effect on total T cells did not require phosphorylation of cladribine by DCK [23], but no data were available on T cell subsets, such as CD8^+^ and CD4^+^ T cells, and NK cells. On the other hand, in proliferation and apoptosis assays, deoxycytidine abrogated the effect induced by cladribine to the levels of the untreated condition, preventing cell proliferation and death. These data indicate that the decrease in immune cell activation by cladribine might be mediated through a different pathway not requiring prodrug activation. In this line, adenosine receptors, expressed on lymphocytes (CD4^+^ and CD8^+^ T and B cells) and NK cells [24], emerge as a possible pathway in the response to cladribine treatment in immune cells, since they are known to modulate immune cell function and can be activated by cladribine [25].

Besides its effects on lymphocytes, cladribine has been shown to have cytotoxic and immunomodulatory effects on innate immune cells, including monocytes and DCs [10,26], which could also contribute to its therapeutic efficacy. Monocytes contribution in MS is well documented [27]. The role of monocytes and their derivatives in presenting antigens to drive immune responses is a key function in the development of MS, in addition to its role in the production of inflammatory cytokines and chemokines [28,29]. Activated monocytes exhibit increased levels of inflammatory molecules, and several reports have demonstrated the effect of MS treatments on the activation status of these cells [30,31,32]. Previous studies have demonstrated that cladribine is cytotoxic on monocytes [10,33]; consequently, a fixable viability dye was included, in order to exclude dead cells in the activation functional assay. In our hands, a cytotoxic effect was also observed in isolated monocytes treated in vitro at the mean concentration found in the blood of cladribine-treated patients. Moreover, the in vitro treatment significantly decreased the frequency of classical monocytes, and slightly increased the percentage on non-classical monocytes. Previous studies have demonstrated a perturbed balance of circulating monocytes in MS patients, compared with the controls [34,35], and other MS treatments such as glatiramer acetate have shown an increased frequency of non-classical monocytes [36]. Interestingly, classical monocytes are suggested to give rise to non-classical monocytes and the latter are considered a differentiated form of classical monocytes [37]. The possibility that cladribine may induce the switch between monocyte phenotypes remains to be clarified. In addition to the cytotoxic effect and the impact on the frequency of monocyte phenotypes, we also investigated the effects of cladribine on the activation status of monocytes, as determined by the expression of the activation markers CD25, CD80, and HLA-DR. Cladribine significantly reduced the expression of CD25 on classical monocytes, whereas the expression of CD80 and HLA-DR was unchanged. On the other hand, cladribine did not modify the expression of any of the measured activation markers in non-classical monocytes. So far, this is the first study evaluating the immunomodulatory effects of cladribine on monocyte subsets.

DCs have the unique capacity to initiate and regulate immune responses, and thus play a crucial role in autoimmune diseases [38]. Among other relevant functions, DCs are important in priming self-specific T cells, and hence can be another key target for the action of cladribine to mediate its immunosuppressive effects. The moDCs used in this study are comparable to inflammatory DCs, in terms of their phenotype and function [39], and therefore are a suitable system to study the effect of cladribine on DCs. In the conditions used for this experiment, 16 h and 0.1 µM of cladribine, the treatment was not cytotoxic for the cells and, indeed, previous studies have demonstrated that longer incubation periods with cladribine are necessary to induce cell death in DCs [10]. Regarding the effect of the drug on LPS-induced DC maturation, ImDCs responded to LPS with increased expression of the maturation marker CD83, and cladribine did not interfere with this process.

Although our study allows the investigation of different immunomodulatory effects induced by cladribine in several cell populations, a limitation of the study is that all experiments were performed in cells treated with cladribine in vitro. Thus, our findings need to be confirmed ex vivo, in patients treated with cladribine. In this regard, other studies have addressed the ex vivo effects of cladribine, but mainly focus on cell depletion after treatment [9,22,40]. On the other hand, more cell types involved in the disease could have been added to the analysis, for instance, further subcategories of T and B cells as well as regulatory lymphocytes.

According to our current understanding, peripheral immune cells are involved in disease progression, and the modulation of T, B and NK cells by MS treatments have demonstrated efficacy in RR-MS patients (reviewed in [41]). In this line, cladribine has also demonstrated efficacy in the progression of RR-MS, by means of targeting immune cells, however, most of the attributed effects of cladribine on these cells were associated with cell depletion [9,22,40].

In summary, our findings demonstrate that clinically relevant concentrations of cladribine in vitro decreased proliferation, induced apoptosis, and reduced the activation of different cellular subsets of PBMCs. In the process of proliferation and apoptosis, cladribine exerted its immunomodulatory effects in a DCK-dependent manner. On the other hand, cell activation occurs in both DCK-dependent and -independent settings. Finally, our findings concerning the effect on myeloid-derived cells, monocytes and moDCs, showed that cladribine induced cytotoxicity, and impaired the activation of classical monocytes; however, this drug seems to have no major effects on DC maturation. Altogether, these data indicate that cladribine, in addition to its cytotoxic function, can mediate immunomodulation in different immune cell populations, regulating their capacity to proliferate, maturate and activate.

## Data Availability

Data will be made available upon request via email to the corresponding authors (nicolas.fissolo@vhir.org and manuel.comabella@vhir.org).

## Supplementary Materials

The following are available online at https://www.mdpi.com/article/10.3390/cells10123488/s1. Table S1: List of antibodies and clones used to stain human samples. Figure S1: Gating strategy used to study cell proliferation by CFSE decay on PBMCs. Figure S2: Gating strategy used to analyze the apoptosis functional assay on PBMCs. Figure S3: Gating strategy to evaluate the activation of PBMCs. Figure S4: Flow cytometry gate-strategy used to quantify the activation of isolated monocytes. Figure S5: Gating strategy to evaluate DC maturation.
