# Supplementary material for: Immunomodulatory Effects Associated with Cladribine Treatment
Source: Cells. 2021 Dec 10;10(12):3488. doi: 10.3390/cells10123488 (PMC8700070; doi:10.3390/cells10123488)
Supplement: Supplementary file 1 [file cells-10-03488-s001.zip › cells-1424259-supplementary.pdf]

Figure S1: Gating strategy used to study cell proliferation by CFSE decay on PBMCs. (A) Lymphocytes were defined by SSC-Area and FSC-Area. Doublets (FSC-H and FSC-A gating), dead cells (fixable viability stain) and red blood cells (CD235a^{+}) were excluded from the analysis. Proliferation of total lymphocytes (CD45^{+}), T cells (CD3^{+} of the CD45^{+} population), CD4 T cells (CD4^{+}CD8^{-} of the CD45^{+}CD3^{+} population), CD8 T cells (CD4^{-}CD8^{+} of the CD45^{+}CD3^{+} population), NK cells (CD56^{+} of the CD45^{+}CD3^{-} population) and B cells (CD19^{+} of the CD45^{+} population) was analyzed. (B) A representative scheme of CD45^{+} proliferating cells based on CFSE decay is shown. RBC: red blood cells.

Figure S2: Gating strategy used to analyze the apoptosis functional assay on PBMCs. (A) Lymphocytes were gated on the basis of their FSC-A and SSC-Area. Doublets (FSC-H and FSC-A gating) and red blood cells (CD235a^{+}) were excluded from the analysis. Apoptosis of total lymphocytes (CD45^{+}), T cells (CD3^{+} of the CD45^{+} population), CD4 T cells (CD4^{+}CD8^{-} of the CD45^{+}CD3^{+} population), CD8 T cells (CD4^{-}CD8^{+} of the CD45^{+}CD3^{+} population), NK cells (CD56^{+} of the CD45^{+}CD3^{-} population) and B cells (CD19^{+} of the CD45^{+} population) was analyzed. (B) A representative panel to quantify apoptosis based on Annexin V/7AAD staining is depicted for CD45^{+} cells. RBC: red blood cells.

Figure S3: Gating strategy to evaluate the activation of PBMCs. (A) Lymphocytes were defined by SSC-Area and FSC-Area. Doublets (FSC-H and FSC-A gating), dead cells (fixable viability stain) and red blood cells (CD235a^{+}) were excluded from the analysis. Activation of total lymphocytes (CD45^{+}), T cells (CD3^{+} of the CD45^{+} population), CD4 T cells (CD4^{+}CD8^{-} of the CD45^{+}CD3^{+} population), CD8 T cells (CD4^{-}CD8^{+} of the CD45^{+}CD3^{+} population), NK cells (CD56^{+} of the CD45^{+}CD3^{-} population) and B cells (CD19^{+} of the CD45^{+} population) was analyzed. (B) A representative scheme of CD45^{+}CD69^{+} activated cells is shown. RBC: red blood cells.

Figure S4: Flow cytometry gate-strategy used to quantify the activation of isolated monocytes. (A) Monocytes were defined by SSC-Area and FSC-Area. Doublets (FSC-H and FSC-A gating) and dead cells (fixable viability stain) were excluded from the analysis. Monocyte subsets were identified based on the expression of CD14 and CD16; classical monocytes (CD14^{++}CD16^{-}) and non-classical (CD14^{+}CD16^{++}) monocytes were discriminated in a CD14 vs CD16 plot. (B) Representative schemes for each of the activation markers, CD80, CD25, and HLA-DR vs. FSC-A in classical or non-classical monocytes are displayed.

Figure S5: Gating strategy to evaluate DC maturation. DCs were defined by SSC-Area and FSC-Area. Doublets (FSC-H and FSC-A gating) and dead cells (fixable viability stain) were excluded from the analysis. Alive cells were represented in a density plot CD14 vs. FSC-A, and moDCs differentiated from monocytes were gated (CD14^{-}). mDCs were quantified based on the expression of CD209 (DCsign) vs. CD83 (maturation marker).

Supplementary Figure S1: Gating strategy – Proliferation assay

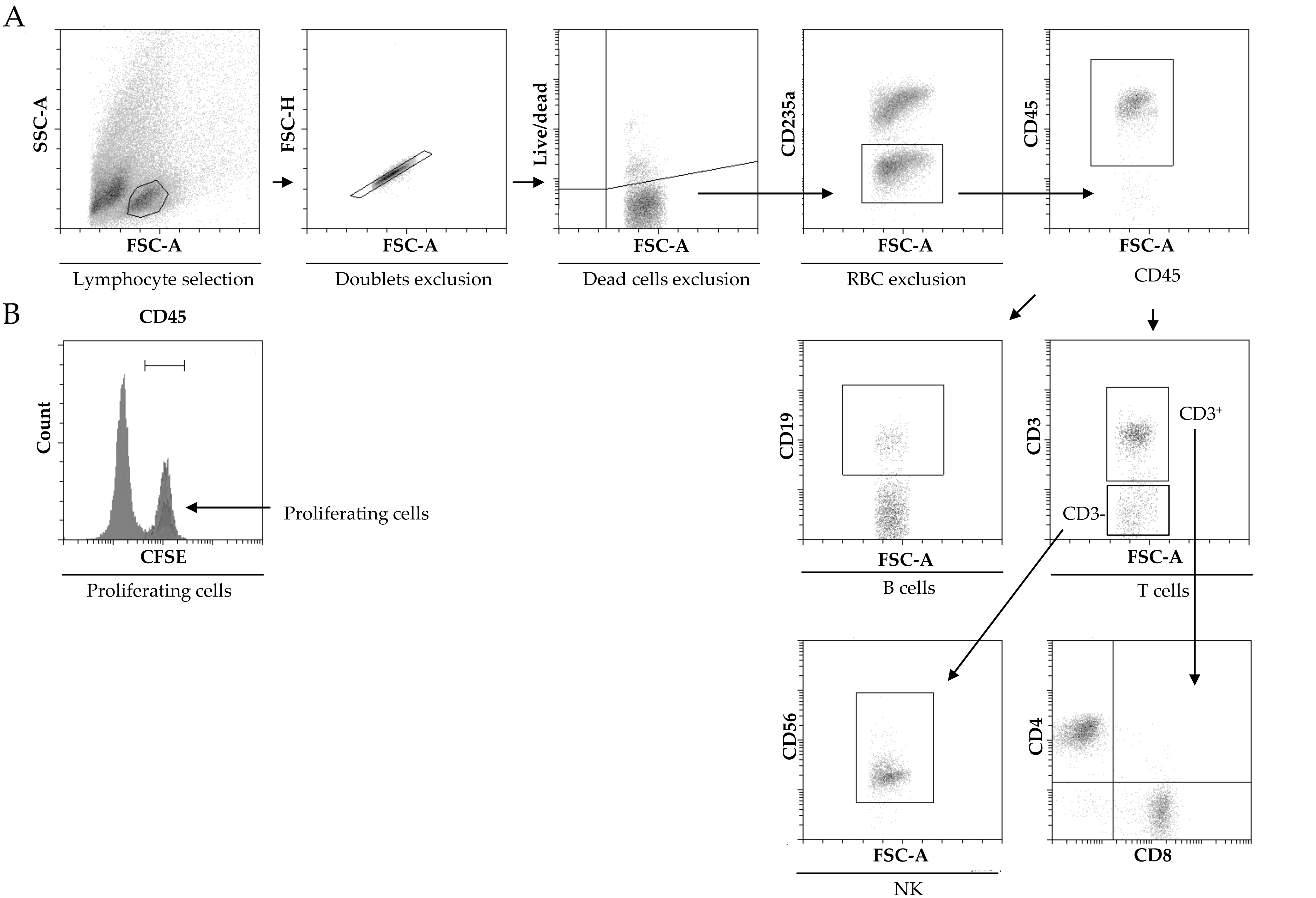

Supplementary Figure S2: Gating strategy – Apoptosis assay

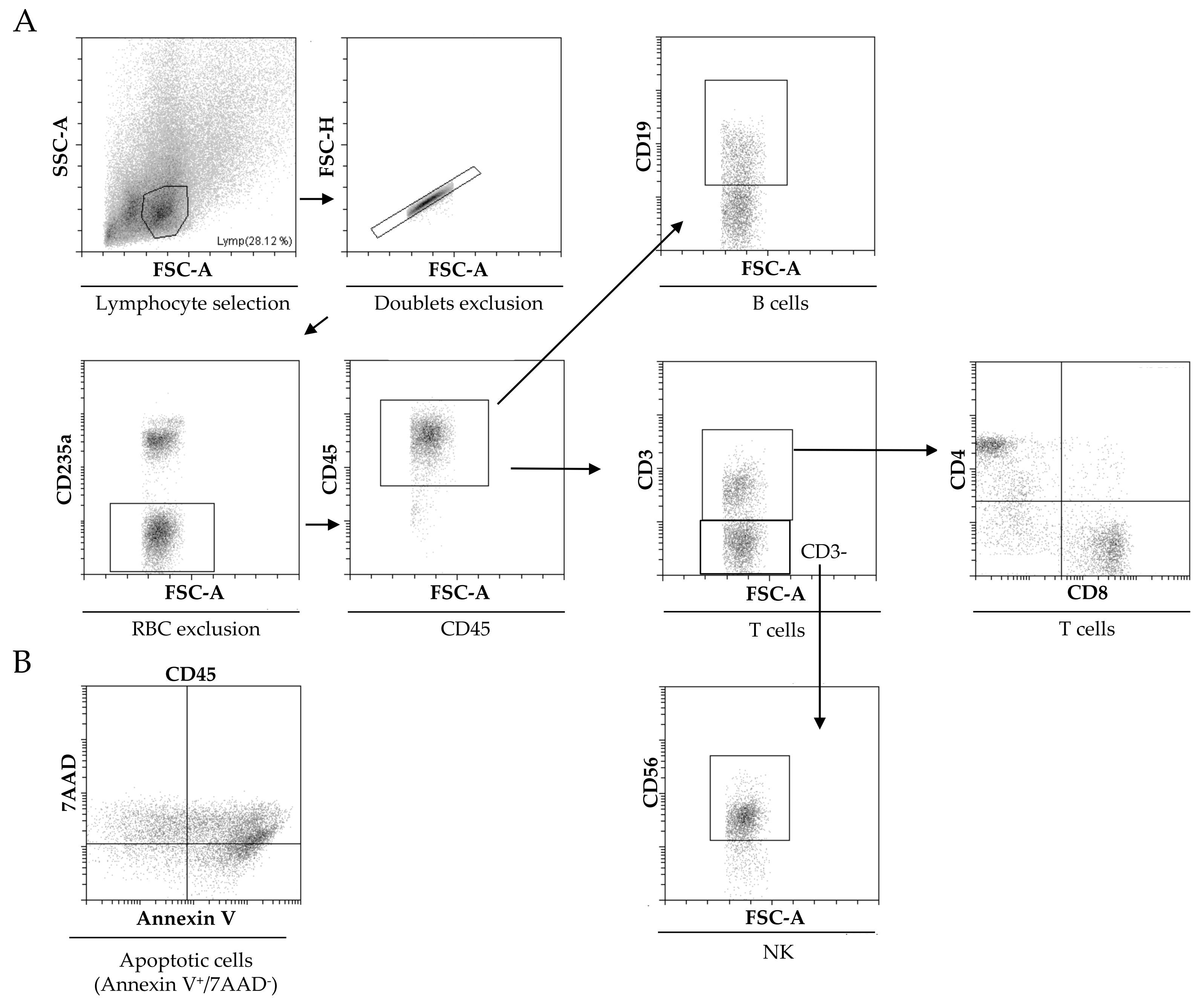

Supplementary Figure S3: Gating strategy – Activation assay

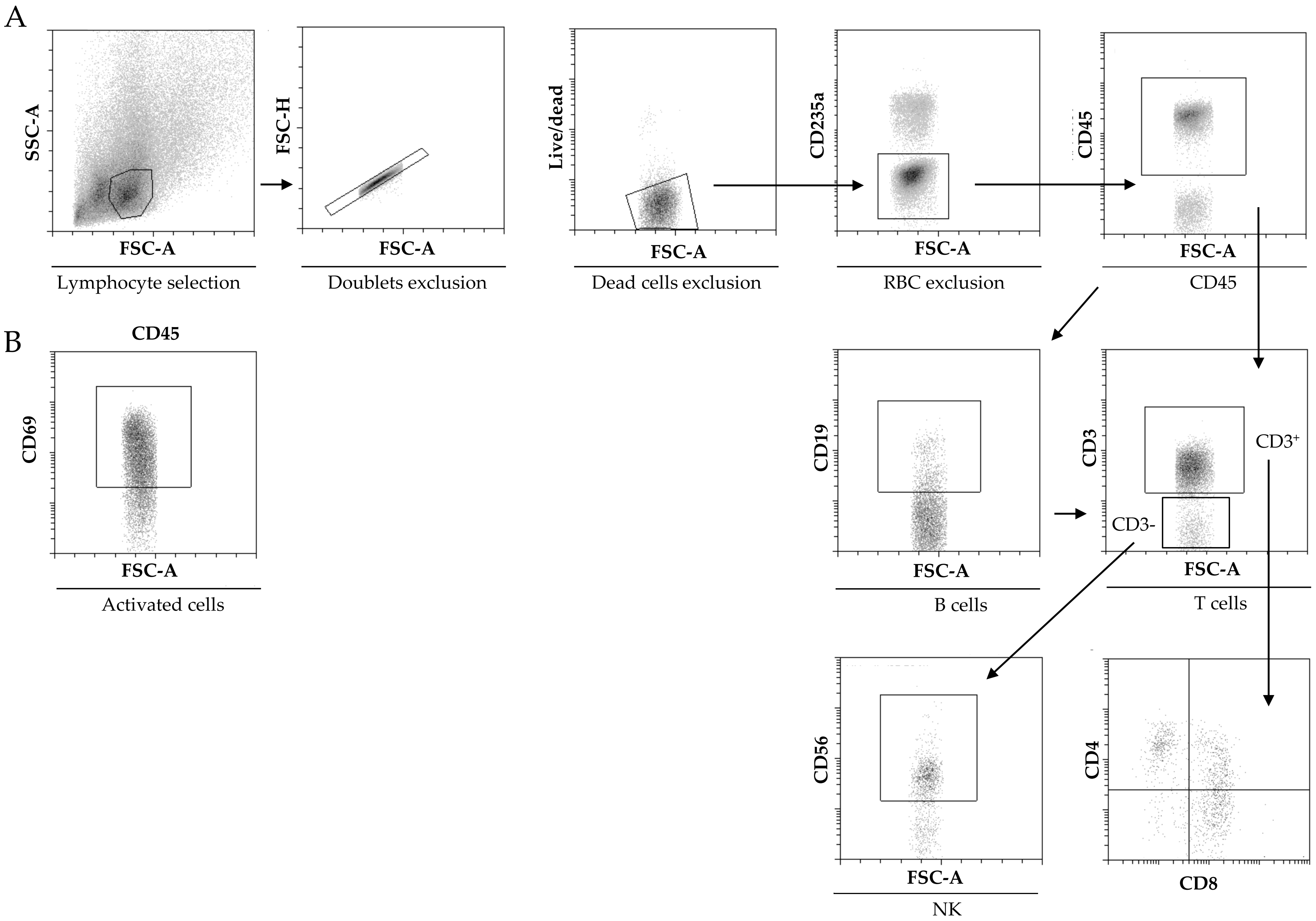

Supplementary Figure S4: Gating strategy – Monocyte activation

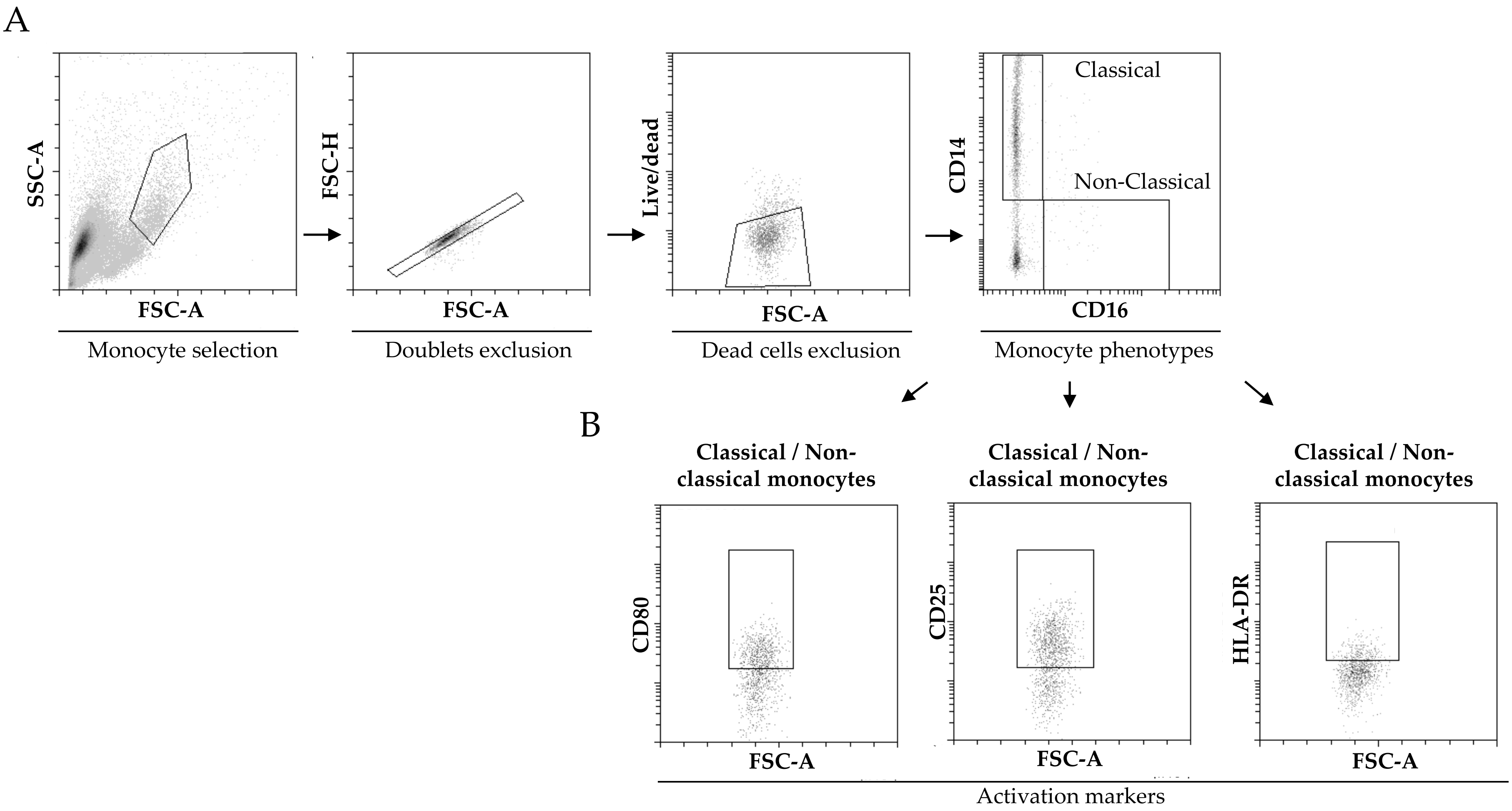

Supplementary Figure S5: Gating strategy – Dendritic cell maturation

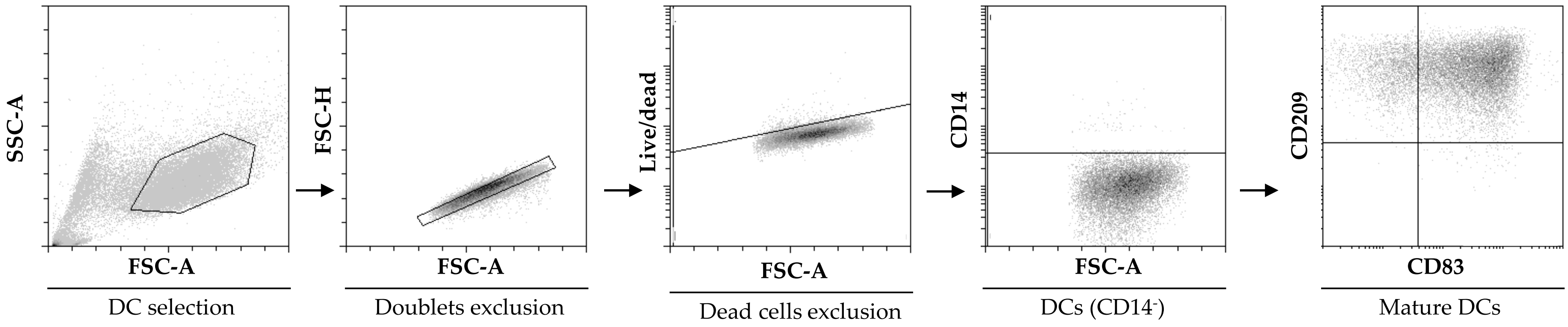

Supplementary Table S1: Antibody Panels

Antibody panel used for immune-cell staining of PBMC in the apoptosis assay (Methods 2.4.1).

Target	Clone	Fluorochrome	Catalog	Vendor	Purpose
CD3	SK7	FITC	344804	Bio	Lineage
CD4	SK3	Alexa Fluor 700	344622	Bio	
CD8	SK1	BV510	563919	BD	
CD19	SJ25C1	APC-H7	560177	BD	
CD45	HI30	BV605	564047	BD	
CD56	R19-760	PE	563238	BD	
CD235a	GA-R2	PE-Cy7	563666	BD	Erythrocyte exclusion
7AAD			559925	BD	Necrosis
Annexin V	R19-760	APC	550474	BD	Apoptosis
Fixable Viability Dye		eF450	65-0863-14	eBio	Dead cells exclusion

BD = BD Biosciences, Bio = BioLegend, eBio = eBioscience

Antibody panel used for immune-cell staining of PBMC in the CFSE-proliferation assay (Methods 2.4.2).

Target	Clone	Fluorochrome	Catalog	Vendor	Purpose
CD3	SK7	APC	344812	Bio	Lineage
CD4	SK3	Alexa Fluor 700	344622	eBio	
CD8	SK1	BV510	563919	BD	
CD19	SJ25C1	APC-H7	560177	BD	
CD45	HI30	BV605	564047	BD	
CD56	R19-760	PE	563238	BD	
CD235a	GA-R2	PE-Cy7	563666	BD	Erythrocyte exclusion
CFSE			C34554	TF	Proliferation
Fixable Viability Dye		eF450	65-0863-14	eBio	Dead cells exclusion

BD = BD Biosciences, Bio = BioLegend, eBio = eBioscience, TF = ThermoFisher

Antibody panel used for immune-cell staining of PBMC in the activation assay (Methods 2.4.3).

Target	Clone	Fluorochrome	Catalog	Vendor	Purpose
CD3	SK7	FITC	344804	Bio	Lineage
CD4	SK3	PerCP-eFluor 710	46-0047-42	eBio	
CD8	SK1	BV510	563919	BD	
CD19	SJ25C1	APC-H7	560177	BD	
CD45	HI30	BV605	564047	BD	
CD56	R19-760	PE	563238	BD	
CD235a	GA-R2	PE-Cy7	563666	BD	Erythrocyte exclusion
CD69	FN50	APC	555533	BD	Activation
Fixable Viability Dye		eF450	65-0863-14	eBio	Dead cells exclusion

BD = BD Biosciences, Bio = BioLegend, eBio = eBioscience

Antibody panel used for immune-cell staining of monocytes in the activation assay (Methods 2.5).

Target	Clone	Fluorochrome	Catalog	Vendor	Purpose
CD14	MφP9	PE-Cy™7	562698	BD	Lineage
CD16	CB16	FITC	11-0168-42	eBio	
CD25	BC96	BV421	302630	Bio	Activation
CD80	L307.4	PE	557227	BD	
HLA-DR	L243	PerCP	347402	BD	
Fixable Viability Dye		eF450	65-0863-14	eBio	Dead cells exclusion

BD = BD Biosciences, Bio = BioLegend, eBio = eBioscience

Antibody panel used for immune-cell staining of ImDC and mDCs in the differentiation assay (Methods 2.2).

Target	Clone	Fluorochrome	Catalog	Vendor	Purpose
CD14	Tük4	FITC	130-093-567	Milt	Lineage
CD209	DCN-47.5.4	PE	130-093-567	Milt	
CD83	HB15	APC	130-093-567	Milt	Maturation

Milt = Miltenyi
